# Alpha-Glucosidase Inhibitory Peptides: Sources, Preparations, Identifications, and Action Mechanisms

**DOI:** 10.3390/nu15194267

**Published:** 2023-10-05

**Authors:** Han Lu, Tiantian Xie, Qi Wu, Zuomin Hu, Yi Luo, Feijun Luo

**Affiliations:** 1 Hunan Key Laboratory of Grain-Oil Deep Process and Quality Control, Central South University of Forestry and Technology, Changsha 410004, China; lu18365138735@163.com (H.L.); xtt04160530@163.com (T.X.); 17663231179@163.com (Q.W.); huzuomin97100214@163.com (Z.H.); 2 Hunan Key Laboratory of Forestry Edible Resources Safety and Processing, Central South University of Forestry and Technology, Changsha 410004, China; 3 Department of Gastroenterology, Xiangya School of Medicine, Central South University, Changsha 410008, China; lyzndx0810@163.com

**Keywords:** α-glucosidase inhibitory peptide, source, mechanism, bioavailability

## Abstract

With the change in people’s lifestyle, diabetes has emerged as a chronic disease that poses a serious threat to human health, alongside tumor, cardiovascular, and cerebrovascular diseases. α-glucosidase inhibitors, which are oral drugs, have proven effective in preventing and managing this disease. Studies have suggested that bioactive peptides could serve as a potential source of α-glucosidase inhibitors. These peptides possess certain hypoglycemic activity and can effectively regulate postprandial blood glucose levels by inhibiting α-glucosidase activity, thus intervening and regulating diabetes. This paper provides a systematic summary of the sources, isolation, purification, bioavailability, and possible mechanisms of α-glucosidase inhibitory peptides. The sources of the α-glucosidase inhibitory peptides were introduced with emphasis on animals, plants, and microorganisms. This paper also points out the problems in the research process of α-glucosidase inhibitory peptide, with a view to providing certain theoretical support for the further study of this peptide.

## 1. Introduction

According to the International Diabetes Federation, as of 2021, about 537 million people worldwide have diabetes, with a prevalence of more than 10% [1]. By 2045, the number of diabetes patients in China will reach an unprecedented 174 million, ranking first in the world. Diabetes can lead to severe injury, dysfunction, and failure of multiple organs and tissues, with progressive metabolic complications, such as neuropathy, retinopathy [2,3], kidney disease [4], and cardiovascular disease [5]. There are three types of diabetes: type 1 diabetes, type 2 diabetes, and gestational diabetes. About 80% of diabetes is type 2 diabetes and their pharmacological treatments focus on increasing insulin action or inhibiting carbohydrate digestive enzymes such as glycosidase inhibitors, thereby reducing blood glucose concentration [6,7]. Therefore, the control of blood glucose is a key step in the managements and treatments of diabetes.

Glycosidases are enzymes that play an important role in glucose metabolism. They can hydrolyze glucoside bonds and release monosaccharides. Glucosidase are the most important enzymes in the glycosidase family and their main functions are to hydrolyze glucosidase bonds and release glucose. Glucosidase can be divided into α-glucosidase and β-glucosidase according to the break mode of the glycoside bond. α-Glucosidase (E.C.3.2.1.20) includes maltase, isomaltase, glucoamylase, and sucrose and can bind to the membrane of small intestinal epithelial cells [8]. The function of α-glucosidase is to break the α-glucoside bond and release α-glucose from the non-reducing end of oligosaccharides. The α-amylase secreted by the salivary glands and pancreas can hydrolyze carbohydrates from food into oligosaccharides, which are then broken down into absorbable glucose by α-glucosidase in the small intestine [9]. Glucose is absorbed into the bloodstream by epithelial cells in the upper small intestine, resulting in a postprandial glucose elevation. α-Glucosidase inhibitors can competitively inhibit the activity of small intestinal α-glucosidase and delay or inhibit the absorption of glucose in the small intestine, thereby effectively reducing blood glucose concentrations [10]. Recent studies have found that active peptides have many biological functions [11,12,13,14,15,16]. Some active peptides can obviously inhibit the activity of α-glucosidase; it suggests that α-glucosidase inhibitory peptide has potential application in the prevention and treatment of type 2 diabetes.

Bioactive peptides can be classified into natural peptides and synthetic peptides according to their sources [17]. Natural peptides are further divided into endogenous and exogenous active peptides [18]. Endogenous bioactive peptides are produced by human tissues and organs, while exogenous bioactive peptides are produced by proteases degradation and microbial fermentation. In this paper, the sources, preparations, identifications, and action mechanisms of α-glucosidases, as well as its bioavailability and treatment of diabetes, are reviewed, which will provide valuable references for the investigators in this field.

## 2. Production, Purification, and Characterization of Bioactive Peptides

### 2.1. Conventional Approach

The main preparation methods of α-glucosidase inhibitory peptides include enzymatic hydrolysis, microbial fermentation, and chemical methods [19,20]. Chemical synthesis can synthesize peptide chains with specific structures and sequences, but the cost is high in mass production and it is easy to cause environmental pollution [21]. The microbial fermentation method utilizes microbial metabolism-produced enzymes to hydrolyze the proteins in the fermented raw materials. It has the advantages of high hydrolytic activity and low cost and does not need to separate and purify the enzyme. The disadvantage is that the reaction process is not easy to control and is susceptible to microbial contamination. Among them, enzymatic hydrolysis is the most widely used method, and we should pay attention to select suitable enzymes when we hydrolyze the protein, which can extract a short peptide chain and improve the digestibility, so as to retain the nutritional value of the food to the maximum.

**Figure 1:**
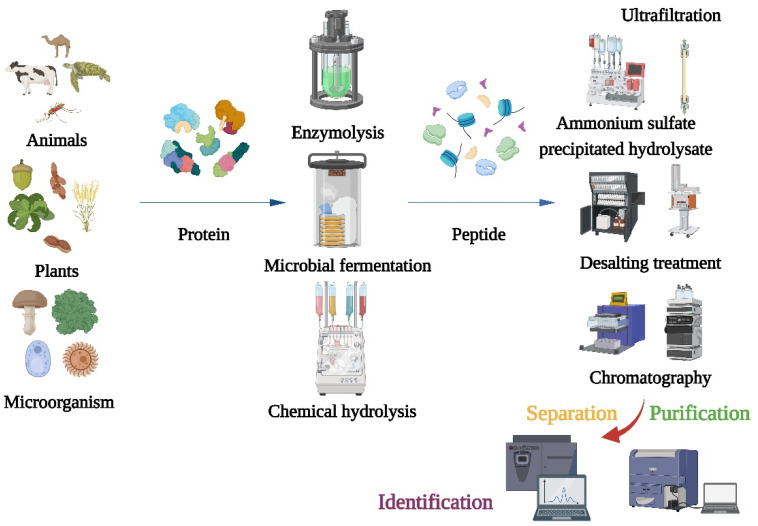
Flow chart of traditional method for preparation, isolation, purification, and identification of α-glucosidase inhibitory peptide.

After producing α-glucosidase inhibitory peptides, different separation and purification techniques should be adopted to obtain the purified products (Figure 1). Purifications of bioactive peptides are the prerequisite for identifying the structures of peptides and detecting their activities. The purifications of peptides are mainly based on their molecular size, charge, polarity, solubility, and other physical chemistry properties [22]. Meanwhile, the combination of multidimensional chromatography and various separation methods can obtain better separation results. The first step of purification is usually precipitation of the hydrolysate with ammonium sulfate, followed by desalting. Because α-glucosidase inhibitory peptides are usually small, the hydrolyzed products are typically truncated to 1 kDa, 3 kDa, 5 kDa, and 10 kDa for membrane filtration [23,24]. After filtrations, different chromatography techniques such as size-exclusion chromatography (SEC), ion exchange chromatography, gel chromatography, and reversed-phase high-performance liquid chromatography (RP-HPLC) have been used to purify different peptides [25,26]. And, both the purities and sequences of the peptides can be identified using mass spectrometry (MS). In recent years, more and more advanced techniques have been used for peptide identifications such as quadrupole time-of-flight mass spectrometry (Q-TOFMS), matter-assisted laser desorption time-of-flight mass spectrometry (MALDI-TOF), and electrospray ionization mass spectrometry (ESI-MA) [27,28,29]. Liquid-phase mass spectrometry (LC-MS) is the most commonly used method to identify peptide sequences. After purification, the collected fractions should be freeze-dried and their α-glucosidase inhibitory activities should be determined. Meanwhile, the molecular mass, amino acid composition, and sequence of the α-glucosidase inhibitory peptide can be determined using sodium dodecyl sulfate-polyacrylamide gel electrophoresis (SDS-PAGE) and protein sequencing.

Traditional methods have many problems in the analytical process: (1) Some peptides have very low levels in the mixture or interact with other components, which make their isolations and purifications very difficult; (2) Some highly active peptides may not be detected using conventional methods, especially those that target novel structures or mechanisms; (3) More experimental steps may be required, which will consume time and resources and, thus, increase the cost of investigation, especially when peptides are isolated and characterized on a large scale; (4) When large numbers of peptides need to be isolated from complex protein mixtures, traditional methods may not be appropriate, and there may be efficiency and scalability issues when purifying peptides on a large scale. To overcome these problems, new technologies and methods have emerged in recent years [30,31,32,33,34]. For example, the development of methods such as proteomics, mass spectrometry imaging, high-throughput sequencing, and computer-assisted screening provide more efficient, accurate, and cost-effective methods for analyzing and discovering bioactive peptides.

### 2.2. Bioinformatics-Assisted Methods

Computer research is also known as “computer methods” or “bioinformatics”. Bioactive peptides are studied using databases and bioinformatics tools. It accelerates the process of discovery and design of α-glucosidase inhibitory peptides by predicting peptide structure, function, and interactions. These predictions are derived and analyzed by computers from existing experimental data. Bioinformatics technology plays an important role in the study of α-glucosidase inhibitory peptides and has great potential to further improve the current state of research. An increasing number of databases are being created and used and bioinformatics tools allow for a range of studies [35]: (1) A large amount of bioactive peptide structural data was collected in the structural database, which can analyze the relationship between structural patterns and functional features of peptides. These analytical results are helpful to understand the similarities and differences among different bioactive peptides, reveal the relationship between structures and activities, and investigate the optimization of their the structures to improve their activities and stabilities. (2) Using structural information from the bioactive peptide database can help researchers design new bioactive peptide molecules using computational simulations. This simulation technique focuses on optimizing peptide design and synthesis strategies by assessing the stabilities or affinities of peptides to their targeting sites. (3) The bioactive peptide database collects a large number of known bioactive peptides and their related data. By utilizing methods such as machine learning and data mining, prediction models can be built to identify and predict potentially bioactive peptide molecules. This prediction can help researchers quickly screen and evaluate the activity of candidate peptides among a large number of peptide samples, thus improving the preparation efficiency and success rate. The application of bioinformatics in the field of peptides provides a great opportunity for the study of α-glucosidase inhibitory peptide. The known α-glucosidase inhibitory peptides have a large amount of structure, sequence, and activity data, which can be used to establish screening procedures and prediction models effectively and to screen effective peptides or design new peptides more quickly. This will shorten the experimental process and reduce costs.

**Figure 2:**
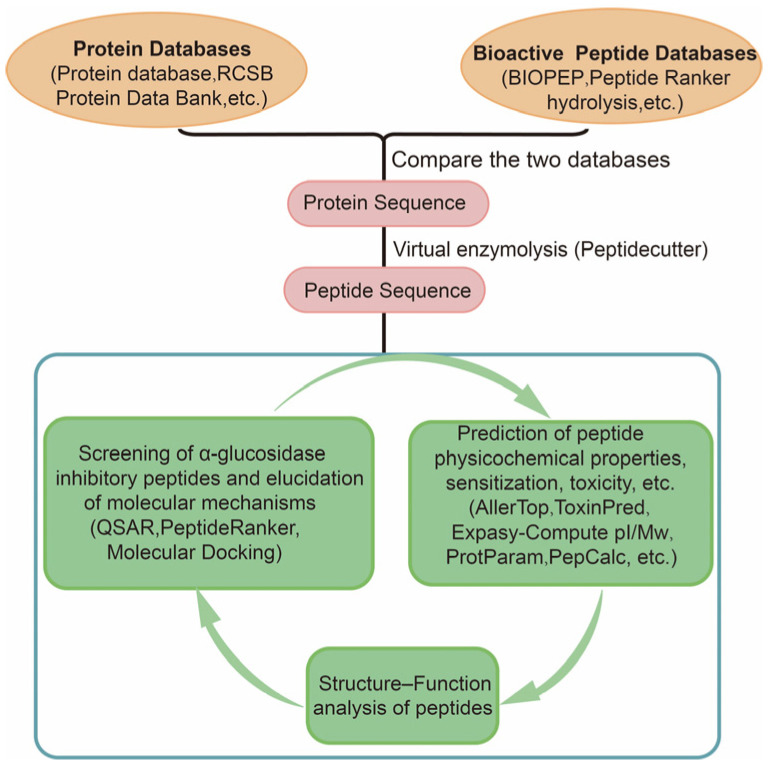
In silico approach combined with bioinformatics screening of α-glucosidase inhibitory peptides.

There are several steps involved in assessing or predicting the role of α-glucosidase inhibitory peptides using bioinformatics (Figure 2). The first step requires a comparison using data from two databases (protein and peptide sequences), where protein databases include the Protein database, RCSB Protein Data Bank, ExPASy, UniProtKB, etc. The bioactive peptide databases include BIOPEP, Peptide Ranker, Peptidecutter, EnzymePredictor, and so on. Potential sources of bioactive peptides can be obtained by evaluating protein sequences and protein sources of peptides with known sequence peptides. Peptidecutter can perform computer simulations of enzymatic hydrolysis, which predicts where specific substances will cleave a protein sequence under known conditions. Computer simulation of enzymatic hydrolysis can greatly improve the efficiency of active peptide screening, optimize peptide sequence design based on simulation results, and select more promising candidate peptides. In most cases, it is necessary to evaluate peptide digestion in the organism. The BIOPEP database is used to assess the likelihood of the designed peptide sequence being hydrolyzed by peptidases within gastrointestinal digestion. In addition, for known peptide sequences, the potential biological activity can be further predicted using quantitative structural relationships (QSAR), the online tool PeptideRanker, and molecular docking. Basic peptide properties including allergenicity, toxicity, molecular weight, isoelectric point, stability, hydrophilicity, physical and chemical properties can be analyzed using various online analytical tools such as AllerTop, ToxinPred, AlgPred, EPIMHC, SPRALLER, Expasy-Compute pI/Mw, ProtParam, and PepCalc [36]. These analyses help to evaluate peptide characterization, and accelerate the screening and design process of bioactive peptides.

## 3. Source and Structural Characteristics of α-Glucosidase Inhibitory Peptide

**Figure 3:**
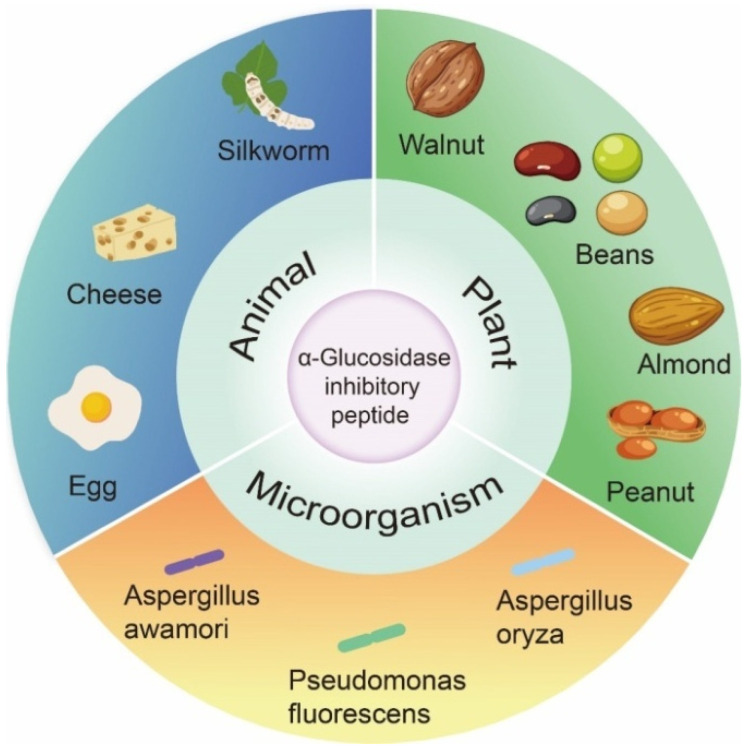
Origin of α-glucosidase inhibitory peptides.

In past studies, the focus of α-glucosidase inhibition peptides has shifted to identification and characterization, which are mainly derived from the partial hydrolysis of proteins from plants, animals, and microorganisms (Figure 3). As shown in Table 1, many bioactive peptides with α-glucosidase inhibitory effects have been identified. Of these peptides, two are cyclic peptide. The length of the 96 α-glucosidase inhibitory peptides ranged from 2 to 16 aa. The number of peptides composed of two to eight amino acids accounted for 90.63% of the total. The most frequently studied peptides were tetrapeptides (16) and pentapeptides (17).

### 3.1. Animal Origin

The proteins in dairy products have high nutritional value. They also contain a significant amount of animal-derived peptides and are considered to be a high-quality source of bioactive peptides. Milk-derived proteins are a precursor of bioactive peptides with various physiological functions. The main protein in milk is casein, which is around 80% of the total amount. Casein is structurally diverse with many sites exposed so that many proteases break down these proteins to form functional bioactive peptides. Numerous studies have shown that milk hydrolysates such as camel milk, cow milk, and goat milk have significant inhibitory effects on α-glucosidase. Casein hydrolysates extracted from bovine and camel milk were found to have a strong inhibitory effect on α-glucosidase, with IC_50_ values of 1.04 mg/mL and 0.59 mg/mL, respectively; LPTGWLM, MFE, and GPAHCLL were the most effective α-glucosidase inhibitory peptides [37]. Similarly, 196 peptides were identified from camel whey protein hydrolysates and 15 potential functional peptides were discovered using computer simulation [38]. Among them, CCGM and MFE were identified as α-glucosidase inhibitory peptides. In addition, due to their high binding sites and binding probability to target enzymes, some novel peptides such as PAGNFLMNGLMHR, PAVACCLPPLPCHM, PAGNFLPPVAAAPVM, and MLPLMLPFTMGY from camel whey protein hydrolysate were identified as potential inhibitors of α-amylase and α-glucosidase [38]. As a source of nutritionally balanced protein, eggs are another ideal source for obtaining α-glucosidase. Yu et al. [39] hydrolyzed egg white protein with alkaline protease and measured the inhibitory activity of the purified peptide. The results showed that RVPSLM and TPSPR had the highest inhibitory activity of α-glucosidase. The IC_50_ values of them were 23.07 μmol/Lol/L and 40.02 μmol/Lol/L, respectively. The new peptides, KLPGF (IC_50_ = 59.50 ± 5.70 μmol/Lol/L) and NVLQPS (IC_50_ = 100.00 ± 5.70 μmol/Lol/L), were identified from albumin with α-glucosidase inhibitory activity. Although KLPGF had a higher inhibitory effect on α-amylase than previously reported RVPSLM, it had a lower inhibitory effect on α-glucosidase [40]. Zambrowicz et al. [27] hydrolyzed the egg yolk protein by-products with pepsin to produce a novel peptide VTGRFAGHPAAQ with α-glucosidase inhibitory activity (IC_50_ = 365.40 μg/mL). Zambrowicz et al. [41] used Asian pumpkin (*Cucurbita ficifolia*) protease, to isolate peptides from egg yolk protein by-products and the obtaining peptide LAPSLPGKPKPD had α-glucosidase inhibitory activity with IC_50_ = 1065.60 mmol/L. LAPSLPGKPKPD and the above-mentioned peptides share common features: the presence of P, S, and L residues in their sequences. It can be assumed that the presence of R residues at the C or N terminus of the peptide chain may result in high levels of α-glucosidase inhibition. Cheese, as an extremely popular dairy product, is rich in nutrients. It also is a potential raw material for the production of bioactive peptides. During cheese ripening, casein is able to produce small bioactive peptides via the action of rennet, proteases of secondary microflora, and peptidases [75]. Researchers have started to focus on finding effective glucosidase inhibitory peptides from cheeses, such as Parmigiano Reggiano cheese [42] or fermented rubing cheese [43]. The above studies have shown that proteins in foods such as dairy products and eggs and the bioactive peptides in their hydrolysis products have the potential to inhibit α-glucosidase and could be widely used in the development of functional foods.

Notably, some peptides composed of amino acids such as Gly, Ser, Glu, Tyr, Arg, Phe, and Pro are considered to be α-glucosidase inhibitors. Lee et al. [44] identified the tripeptides GEY and GYG with α-glucosidase inhibitory activities (IC_50_ of 2.70 and 1.50 mg/mL). Zhang et al. [45] used the quantitative structure–activity relationship screening method and silkworm protein database to identify α-glucosidase inhibitory peptides. Four peptides that have α-glucosidase inhibition effects were obtained and they were QPGR (IC_50_ of 65.80 mol/L), SQSPA (IC_50_ of 20.00 mol/L), QPPT (IC_50_ of 560.0 mol/L), and NSPR (IC_50_ of 205.00 mol/L). QPGR could form hydrogen bonds with Lys776 in the active site ofα-glucosidase and SQSPA had potential interactions with Arg520, Lys519, and Asp777 of α-glucosidase. Compared with QPGR, QPPT and NSPR only form a single bond with Lys776 of α-glucosidase. In the three-dimensional structure, Lys776 is located at the edge of the α-glucosidase activity pocket and may be a key target for these peptides to inhibit α-glucosidase activity [45]. SEDSSEVDIDLGN was a new peptide derived from sericin, and it had been found to non-competitively bind α-glucosidase by hydrogen bonding forces or van der Waals forces. The binding might be dominated by the Asn and Ser side chains which were contained in SEDSSEVDIDLGN [46,76]. In addition, CSSV (IC_50_ = 206.00 μg/mL), SAAP (IC_50_ = 66.90 μg/mL), PGGP (IC_50_ = 63.50 μg/mL), LGGGN (IC_50_ = 42.93 μg/mL), YSFR (IC_50_ = 162.00 μg/mL) from the protein of Giant Salamander, and GPPGPA from skin collagen hydrolysates of Giant Salamander contained higher contents of Gly and Pro [47,48]. This might be why these peptides have a strong inhibitory effect on α-glucosidase. Peptide sequences were identified from edible insects, KVEGDLK, YETGNGIK, AIGVGAIR, IIAPPER, and FDPFPK exhibited the highest enzyme inhibitory activity [49]. The only peptide containing phenylalanine FDPFPK was identified as the most powerful α-glucosidase inhibitor (IC_50_ = 5.95 μg/mL). These results suggest that some peptides composed of specific amino acids can be used as α-glucosidase inhibitors.

Marine biological resources are abundant and the variability of the marine environment makes the composition and arrangement of amino acids unsuitable for terrestrial organisms. It causes them to produce substances with special physiological effects, which are an important source of developing new bioactive substances. Matsui et al. [50] treated sardine muscle hydrolysate with *Bacillus licheniformis* alkaline protease; two peptides were finally screened for their α-glucosidase inhibitory activities: VW (IC_50_ = 22.60 mM) and WYPL (IC_50_ = 3.70 mM). In shellfish, VKP and VKK from *Corbicula fluminea* were effective in inhibiting α-glucosidase activity [51].

### 3.2. Plant Origin

Plants have always been an important source of drug development due to their wide distribution, rich resources, effectiveness, and high safety. Moreover, plant secondary metabolites also can be widely used in pharmaceuticals and other fields. Many researchers have screened α-glucosidase inhibitors from plants and they are a rich source of α-glucosidase inhibitory peptides [77,78,79].

α-Glucosidase inhibitory peptides from various protein-rich plant hydrolysates, such as grains, legumes, and seeds, have been extensively studied. Enzymatic hydrolysis increased the α-glucosidase inhibitory activity of protein hydrolysates [80]. Different proteases have different restriction cutting sites, peptides with different molecular weights, amino acid sequences, and biological activities can be released by cutting peptide bonds at different parts [81]. Commonly used enzymes include alkaline protease, acid protease, pepsin, trypsin, neutral protease, bromelain, flavor protease, etc. Alkaline protease is an endonuclease with a wide range of specificity. It preferentially cleaves the C-terminal peptide bonds of hydrophobic amino acid residues such as Try, Phe, Leu, Ile, Val, Met, etc. This gave it more catalytic sites to cleave peptide bonds in proteins, thus allowing deeper enzymatic cleavage [82]. A longer enzymatic digestion time would result in a higher degree of hydrolysis value of the hydrolyzed product [83]. Trypsin only cleats the C-terminus into Arg and Lys. Flavourzyme are exopeptidases that break the N-terminus of the peptide chain; papain prefers to cleaved peptide bonds between carboxylic acid groups of Lys or Arg and adjacent amino acid residues [84]. Compared with other hydrolysates, the plant protein peptides hydrolyzed by alkaline protease showed the highest α-glucosidase inhibition. Therefore, alkaline proteases has been used to hydrolyze many plant proteins, such as soy protein, *Luffa cylindrical* (L.) M. Roem seed, and *Ginkgo biloba* seed [52,53,85]. For better comparison, previous studies used different commercial enzymes for the enzymatic preparation of α-glucosidase inhibitory peptides under the same conditions. Soybean protein peptides prepared using alkaline protease had the highest α-glucosidase inhibitory activity where compared to those prepared using papain and trypsin. Three new α-glucosidase inhibitory peptides LLPLPVLK, SWLRL, and WLRL were found with IC_50_ values of 237.43 ± 0.52 μmol/Lol/L, 182.05 ± 0.74 μmol/Lol/L, and 165.29 ± 0.74 μmol/Lol/L [52]. Using different proteases to hydrolyze *Luffa cylindrical* (L.) M. Roem seed, the alkaline protease hydrolysate showed the strongest inhibition of α-glucoside, followed by tryptic hydrolysate, with concentration-dependent inhibition of α-glucosidase (IC_50_ of 0.48–0.80 mg/mL) [85]. *Ginkgo biloba* seed was hydrolyzed using alkaline protease and three new peptides with α-glucosidase inhibitory activity were screened: LSMSFPPF, VPKIPPP, and MPGPPSD. LSMSFPPF showed the strongest inhibitory activity (IC_50_ of 454.33 ± 32.45 μmol/Lol/L), followed by MPGPPSD (IC_50_ of 943.82 ± 73.10 μmol/L) and VPKIPPP (IC_50_ of 1446.81 ± 66.98 μmol/Lol/L) [53]. In addition, quinoa is an ancient pseudocereal, and the highest α-glucosidase inhibitory peptide with MW ≥ 3 kDa could be obtained by hydrolyzing quinoa protein with trypsin [86]. The substrate environment often affects the enzymatic hydrolysis process, which changes the amino acid composition and molecular weight of hydrolysates and then affects the inhibitory activity of peptides. Therefore, when preparing α-glucosidase inhibitory peptides, it is necessary to select suitable proteases according to raw materials and experimental conditions and study the optimal enzymatic conditions to obtain a higher number of peptides with high α-glucosidase inhibitory activity.

The candidate peptides were further screened based on molecular weight, amino acid composition, and binding energy to α-glucosidase. Low molecular weight peptides have better stability and higher bioavailability, resulting in better biological function in vivo [87,88,89]. Digestion of soy protein with trypsin to obtain hydrolysates with α-glucosidase inhibitory activity (IC_50_ of 0.27 mg/mL) showed the highest inhibitory activity corresponding to MW < 5 kDa grade fractions and a yield of 76.08% [54]. Two tripeptides GSR (IC_50_ of 20.4 μmol/L) and EAK (IC_50_ of 520.2 μmol/L) were obtained, the inhibitory effect of peptides on α-glucosidase is mainly due to the formation of five strong hydrogen bonds between GSR and His674, Asp518, Arg600, Asp616, and Asp282 in α-glucosidase; four hydrogen bonds were formed between EAK and residues Asp282, Asp518, and Asp616. Asp residues are import targets to inhibit the activity of α-glucosidase [54]. In the easy-to-cook and difficult-to-cook bean hydrolysates, the ultrafiltration fractions with MW < 3 kDa showed the highest inhibition of α-glucosidase (34.4–89.2%) [90]. The subunits of gluten hydrolysates were digested using kiwifruit actinidin. The WGLYH (≤1 kDa) group have the highest inhibitory activities on α-amylase and α-glucosidase [55]. Similarly, chia (*Salvia hispanica*) hydrolysis, rice bran proteins, and black sesame cake also showed that low molecular weight peptides were more likely to react with α-glucosidase inhibitors [91,92,93]. Meanwhile, for longer peptides and large-molecular-weight peptides, binding to the α-glucosidase active site was spatially hindered, resulting in weaker inhibitory activity [94]. This phenomenon may partially explain the weaker inhibitory activity of the fractions with MW > 50 kDa.

According to previous investigations, the peptides with strong α-glucosidase inhibitory activity are short peptides with relative molecular weights less than 1 kDa; this is because lower-molecular-weight peptides can enter the active site of α-glucosidase and bind to it. [28]. However, VVDLVFFAAAK (MW = 1179.4 Da) from black tea protein also exhibited the best α-glucosidase inhibitory ability compared to peptides with MW < 1 kDa [56]. Therefore, MW < 1 kDa was only used as the first screening condition. On the other hand, amino acid composition also contributes significantly to α-glucosidase inhibitory activity. α-Glucosidase inhibitory peptides are usually accompanied by a high degree of hydrophobicity [37,95]. It suggests that hydrophobic amino acid contents in bioactive peptides are closely related with the inhibition activities of α-glucosidases. Quinoa proteins were subjected to simulated gastrointestinal digestion in vitro. Three peptides were isolated and the peptide IQEGGLT (IC_50_ of 109.48 μmol/L) containing three hydrophobic residues showed strong inhibitory activity against α-glucosidase. In contrast, at 250 μmol/L, peptides DKDPYPK (22.16 ± 0.61%) and GEHGSDGNV (30.84 ± 0.69%) showed lower inhibition than IQEGGLT (55.85 ± 0.26%), probably due to their higher hydrophilicity [57]. Quinoa proteins contain high amounts of Gln, Glu, Asp, Asn, Arg, Ser, Leu, and Pro [96]. Ujiroghene et al. [58] identified four antidiabetic peptides from germinated quinoa yogurt drink including VAHPVF, LAHMIVAGA, KLTPQMA, and KSFGSSNI. Among these peptides, LAHMIVAGA and VAHPVF showed significant α-glucosidase inhibitory activity with IC_50_ values of 10.90 mg/mL and 9.00 mg/m. In addition, three novel α-glucosidase inhibitory peptides were isolated from *Paeonia ostii* ‘Feng Dan’ seed protein: YFFM, FFFM, and YYFM [28]. The amino acids of these peptides are all hydrophobic amino acids. The docking study of peptides with α-glucosidase active sites showed that the interactions between peptides and enzymes were mainly hydrogen bonds and π-π superposition [28]. According to previous reports, hydrophobic aliphatic amino acids such as Leu, Ile, Ala, Met, and Pro might promote the antidiabetic ability of food protein hydrolysates or peptides [97]. Both raw grain (PRFM) and cooked grain (PCFM) hydrolysate products exhibited α-glucosidase inhibitory activity. By virtual screening and comparing the fractions with MW < 3 kDa, four peptides including AMFLPGA, TMMMLLP, FFLPQ, and FMLPQ were selected. Then, the composition of inhibitory peptides was determined, respectively. It was found that the proportion of peptides with excellent α-glucosidase binding ability in PCFMH < 3 kDa was higher than in PRFMH < 3 kDa [59]. Among them, peptide TMMMLLP had the highest percentage of hydrophobic amino acids (42.86% for Met, 14.29% for Pro, and 28.57% for Leu). The typical structure of TMMMLLP might make the peptide the typical ligand for binding α-glucosidase [59]. These results may support the notion that peptides containing more hydrophobic amino acids have easier access to the hydrophobic pocket of the α-glucosidase active region, then interacts with its residues.

For the different amino acid composition of peptides, leucine and valine can participate in muscle repair and blood glucose control; proline can improve the hypoglycemic activity of peptide [98]. The peptide GLLGY from rice bran fermentation broth was a non-competitive inhibitor that forms five hydrogen bonds with Asp282, Ser523, Asp616, and His674 of α-glucosidase [25]. GLLGY also maintained excellent α-glucosidase inhibition in the gastrointestinal digestive system [25]. From the structure of α-glucosidase, Asp616 and His674 were the key amino acid residues in the catalytic structural domain, which might be the target of molecular docking of α-glucosidase inhibitors. At the same time, leucine is thought to be critical for enhancing insulin secretion through metabolic allosteric activation and membrane depolarization [99]. Two new α-glucosidase inhibitory peptides were identified from hemp seed protein, with the sequences LR (287.2 Da) and PLMLP (568.4 Da) [60]. Proline and methionine also play important roles in metabolism, nutrition, and immune response, while arginine supplementation can enhance enteral nutrition and improve glucose homeostasis [100,101,102,103]. Similarly, cyclic peptide GFDFILP from the root of *Gypsophila oldhamiana* is also involved in leucine and proline [26]. Thus, proline and leucine in peptides are considered to be important amino acids that act as inhibitors to α-glucosidase individually or synergically. The inhibitory activity of α-glucosidase was also related to the arrangement of amino acids and the structure of peptides [19]. The analysis of the relationship between structure and activity showed that *C*-terminal arginine had a positive effect on the α-glucosidase inhibitory activity. *C*-terminal arginine could improve the stability of peptide–enzyme binding, which ensures the strong binding of peptide and enzyme key active amino acid residues. LDLQR (IC_50_ = 8.59 mM), AGGFR (IC_50_ = 8.66 mM), LDNFR (IC_50_ = 9.21 mM) in wheat germ peptides [61], LRSELAAWSR (IC_50_ = 134.2 μg/mL) in *Spirulina platensis* [62] and, walnut (*Juglans mandshurica* Maxim.)-derived peptide LPLLR [63] showed efficient α-glucosidase inhibitory activity, with the most suitable occurrence position at the *C*-terminal. The analysis of the structure–activity relationship showed that *C*-terminal arginine had a positive effect on α-glucosidase peptide inhibitory activity and could improve the stability of peptide–enzyme binding, ensure the strong binding of peptide to the key active amino acid residues of enzyme, and promote the α-glucosidase inhibitory activity.

The binding energy of plant-derived peptides to α-glucosidase can be predicted by docking. The lower the binding energy required for peptides to bind to α-glucosidase, the easier it is to inhibit the activity of α-glucosidase. Four peptides FYNPAAGR, FFVPPSQQ, PGVLPVAS, and FSYNPQAG were screened from the hydrolysates of hot-pressed peanut meal. Molecular docking indicates that peptides can occupy the active pocket of α-glucosidase through hydrogen bonding, hydrophobic interaction, salt bridges, and π stacking, thus preventing α-glucosidase from forming a complex with substrates. Among these four peptides, PGVLPVAS had the lowest binding energy to α-glucoside, followed by FYNPAAGR, FFVPPSQQ, and FSYNPQAG [64]. Therefore, lower binding energy of peptides than that of acarbose could be used as another screening criterion. The peptide TTGGKGGK (−8.97 kcal/mol) obtained from black bean (*Phaseolus vulgaris* L.) proteins had a higher α-glucosidase inhibitory potential than acarbose. Black soybean peptides inhibit α-glucosidase through hydrogen bonding, polarity, and hydrophobicity. The main binding sites are Asp34, Thr83, and Asn32 [65]. Among the peptides purified from chickpea (*Cicerarietinum* L.) protein hydrolyzates, FGKG showed optimal inhibition of α-glucosidase [66]. Since FGKG exhibited the highest inhibition rate and lowest binding energy (−10.047 kcal/mol), the hydrophobic interactions of FGKG with Leu162-Phe1 and Ala198-Phe1 appeared to contribute significantly to the stabilization of the inhibitor–enzyme complex [66]. Potential peptides were screened from enzymatic hydrolysis products of *Camellia* seed cake through enzymatic hydrolysis. The MS/MS spectra and structures of LLVLYYEY and LLLLPSYSEF showed high α-glucosidase inhibitory activity. The binding energies predicted by LLVLYYEY(IC_50_ = 0.33 mM) and LLLLPSYSEF(IC_50_ = 1.11 mM) were −9.36 and −9.06 kcal/mol, which were lower than −6 kcal/mol [65]. Lineweaver–Burk analysis and molecular docking indicated that peptide LLVLYYEY was the competitive inhibition of α-glucosidase, whereas peptide LLLLPSYSEF exhibited a mixed inhibitory mechanism against α-glucosidase [67]. Ibrahim et al. [104] reported similar binding energy scores for α-glucosidase inhibiting peptides using silica-designed peptide sequences with binding energy scores ranging from −6.3 kcal/mol to −8.7 kcal/mol. Overall, the binding energy of peptides to α-glucosidase can be predicted using molecular docking methods and used as an indicator to assess the α-glucosidase inhibitory activity.

### 3.3. Microbial Origin

As mankind’s knowledge of the world deepens, the vast resources contained in microorganisms are being understood. For peptides, microbiota is a high-quality source that is gradually being exploited by mankind. Due to the short life cycle, fast growth, reproduction, and low culture cost of microorganisms, more and more scholars are focusing on obtaining α-glucosidase inhibitory peptides from microorganism.

Kang et al. [68] found α-glucosidase inhibitors (PFP with a molecular weight of 360.1 kDa) which were obtained from *Aspergillus oryzae* N159-1. The intracellular concentration of this inhibitor reached its highest when the fungus was cultured in trypsin soybean broth medium at 27 °C for 5 days and the IC_50_ value of 3.1 mg/mL for α-glucosidase inhibition. Another study showed an endophytic fungus isolated from the *Acacia nnilotica* was identified as *Aspergillus awamori*, a protein with a molecular mass of about 22 kDa [105]. The purified inhibitor showed mixed-type inhibition of α-glucosidase with an IC_50_ value of 5.625 μg/mL. In addition, three cyclic dipeptides, namely L-Pro-L-Leu, L-Pro-L-Val, and L-Pro-L-Phe, were isolated from *Pseudomonas fluorescein* IB-MR-66e, which had important inhibitory activities against α-glucosidase [69].

## 4. Mechanism of α-Glucosidase Inhibitory Peptide

**Figure 4:**
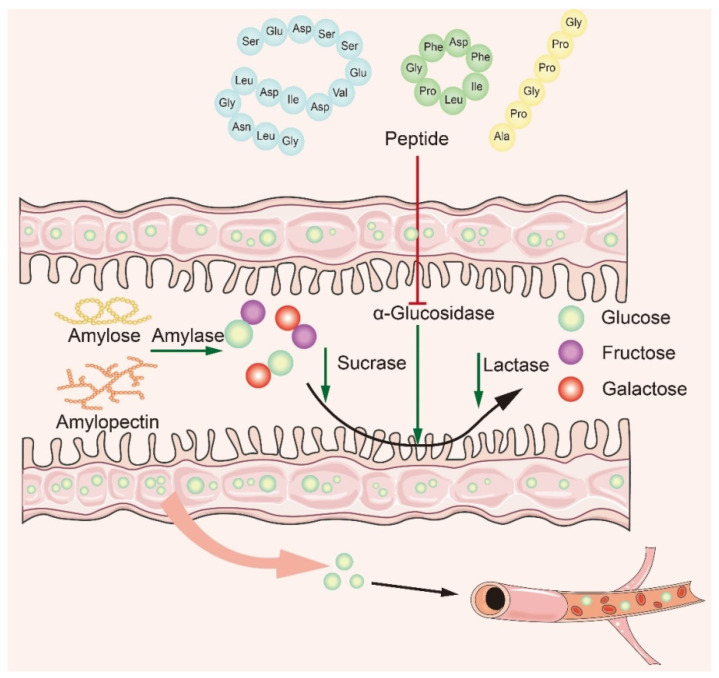
Mechanism of α-glucosidase inhibitory peptides.

Many studies have shown that naturally active peptides could treat type 2 diabetes by lowering blood sugar, but screening for them is a time-consuming process [106,107]. To discover safer and more effective α-glucosidase inhibitory peptides, it is particularly important to explore the interaction mechanism between α-glucosidase inhibitory peptides and α-glucosidase. Based on recent studies, it is concluded that the main mechanism of action is to inhibit α-glucosidase activity in small intestine mucosa (Figure 4). The bioactive peptide mainly binds to oligosaccharides and enzymes, producing competitive inhibition. The peptides act on the enzyme through the hydrophobic effect and hydrogen bonding van der Waals forces, modulating the amino acids at the catalytic sites of the enzyme to affect its original activity and reduce glucose intake [46,108,109]. In recent years, researchers have summarized the structural features and mechanism of α-glucosidase inhibitory peptides: (1) Low-molecular-weight peptides have better α-glucosidase inhibitory activity [110]; (2) Amino acids contain at least a hydroxyl or basic side chain at the N-terminus, with Pro present in the penultimate position at the *C*-terminus [8]; (3) The presence of Arg at the *C*-terminal end improves the stability of the peptide–enzyme binding, ensures strong binding of peptides to key active amino acid residues of the enzyme, and promotes increased inhibitory activity [61]; (4) The higher the content of hydrophobic amino acids, the better the inhibitory activity of α-glucosidase.

Hydrogen bond and hydrophobic interaction play an important role in α-glucosidase inhibition. Qiu et al. [70] used papain to hydrolyze soft-shelled turtle (*Pelodiscus sinensis*) egg to obtain potential anti-diabetic peptides. Furthermore, three oligopeptides, HNKPEVEVR, ARDASVLK, and SGTLLHK, were synthesized, which had strong inhibitory effects on α-glucosidase activity (IC_50_ = 56,195 and 289 μmol/Lol/L, respectively). Li et al. [71] investigated the action mechanism of ARDASVLK and HNKPEVEVR on α-glucosidase using synchronous fluorescence, circular dichroism spectroscopy, and molecular modeling. The amino acid residues that ARDASVLK binds to α-glucosidase mainly include Pro1159, Arg1250, Tyr1251, Gln1254, Glu1258, Trp1355, Trp1369, Gln1372, Arg1377, Asp1526, Phe1559, Ile1587, Gly1588, and Arg1591. HNKPEVEVR interacted with amino acid residues of α-glucosidase as follows: Arg1156, Tyr1251, Trp1355, Leu1367, Asp1368, Trp1369, Asp1370, Phe1427, Asn1429, Val1432, Ser1452, Arg1455, Ser1459, Lys1460, and Ile1587 [71]. Molecular docking and thermodynamic results demonstrated that α-glucosidase interactions with ARDASVLK and HNKPEVEVR were driven by hydrogen bonding and hydrophobic action [71]. The detection by circular dichroism spectroscopy showed that the content of some secondary structures (α-helix and β-sheet) of HNKPEVEVR were altered. Although the cause of these secondary structure changes was unknown, it was clear for minor conformational changes that blocking active center or preventing substrate binding would inactivate the enzyme. Therefore, ARDASVLLK and HNKPEVEVR, which were obtained from turtle eggs, could burst α-glucosidase by forming α-glucosidase-peptide complexes. In this case, the active vacancy of the enzyme was occupied and the conformation was changed, eventually the enzyme activity was diminished. A highly active α-glucosidase inhibitory peptide (MoHpP-2, amino acid sequence: KETTTIVR) with a IC_50_ value of 109.65 μmol/L was identified from moringa seed protein hydrolysate. The binding energy of MoHpP-2 to α-glucosidase was −6.3 kcal^−1^, indicating that MoHpP-2 has a good binding effect. Molecular docking indicated that MoHpP-2 interacted with α-glucosidase protein mainly through hydrogen bonding and hydrophobic force [72]. Another study reported inhibition of α-glucosidase activity via hydrolysis of lupin protein with alkaline and flavor enzymes in six peptides (SPRRF, Fe, R R, PPGIP, LRP, and RPR) [73]. This inhibitory activity prevents carbohydrate hydrolysis by binding to the active site of the enzyme, thus preventing its activation. Molecular docking experiments revealed that the tripeptides RPR and LRP interact with the active pocket of α-glucosidase, which forms a hydrogen bond with the Asp616 residue and interacts with the α-glucosidase catalytic residue via Trp376. The binding energy of LRP was slightly higher than that of RPR [73]. Dipeptide RR interacted with Trp376 and Phe649 via hydrophobic associating (binding energy: −6.4 kcal/mol), and formed hydrogen bonds with residues Asp616 and Asp518. The pentapeptide SPRRF bound to the active site of α-glucosidase with binding energy of −6.6 kcal/mol and formed hydrophobic interactions with the catalytic residue Asp404. FE could form hydrophobic interactions with Phe649, Asp616, and Trp376, and PPGIP used Trp376 for hydrophobic interaction with the α-glucosidase catalytic triad.

The presence of van der Waals forces also affects the activity of α-glucosidase. Three new peptides including LSMSFPPF, VPKIPPPHE, and MPGPPS which had α-glucosidase inhibitory activity were selected from the protein isolates of *Ginkgo biloba* seed cake. There were 12 effector sites of van der Waals force between LSMSFPPF and α-glucosidase [53]. For these sites, Ala454, Phe455, Ala514, Asp440, Thr517, Thr519, Leu525, Asn447, and Arg520 were on α-glucosidase A chain and Leu93, His91, and Lys89 were on the chain B. In addition, VPKIPPP and α-glucosidase also had 12 effector sites of van der Waals force, including Glu377, Thr339, Leu227, Leu300, Pru602, Gly228, Arg400, Tyr389, Glu396, Asn233, Ala229, and Glu231 on chain A. Similarly, 14 van der Waals interactions were observed between MPGPPSD and α-glucosidase, including Asp441 and Asn447 on Chain A; Met6, Asp464, Lys483, Leu93, Leu95, Val47, Asn46, Ala43, Arg456, His459, Pro460, and Gly11 were on chain B [53].

**Figure 5:**
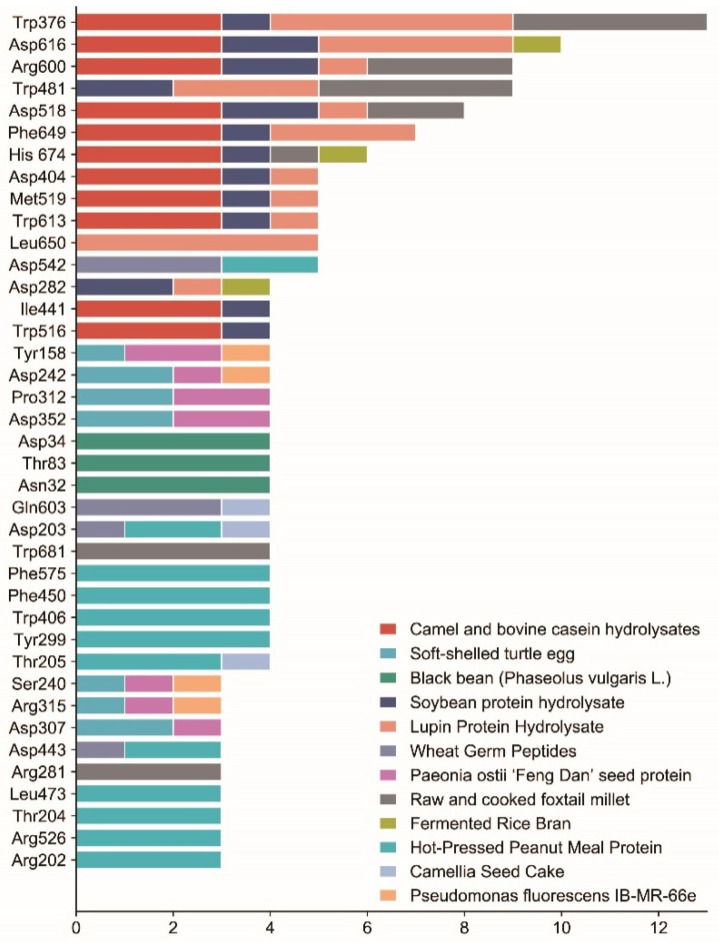
Summary of the key binding sites of α-glucosidase with some typical peptides. The data are based on the peptides mentioned in the literature of this paper that underwent molecular docking. The horizontal coordinate is the number of polypeptides and the vertical coordinate is the key binding site; this graph selects the binding sites with more than 2 polypeptides, different colors represent different sources, and the length of the color represents the number of polypeptides.

For the α-glucosidase inhibitory peptide, it can form hydrogen bonds with the active site of the α-glucosidase which makes the binding more stable. At the same time, the inhibitory peptide interacts with the hydrophobic part of the enzyme to enhance the affinity between the inhibitory peptide and enzyme; the stability of the binding site has also been enhanced. This ultimately prevents the enzyme from binding to oligosaccharides, thereby effectively inhibiting changes in blood glucose. Van der Waals force interactions between α-glucosidase inhibitory peptides and enzymes can affect the binding and dissociation kinetics of inhibitory peptides, thus regulating enzyme activity, and some inhibitory peptides can improve the affinity and selectivity of inhibitory peptides by enhancing van der Waals force interactions with enzymes. The binding sites of the α-glucosidase inhibitory peptides described above are mostly similar. Table 1 lists the potential binding sites of α-glucosidase inhibitory peptides according to their docking structures. Molecular docking techniques can predict binding sites and potential effects, as well as elucidate key structural features. Therefore, molecular docking is important for understanding the mechanisms of α-glucosidase inhibitory peptides. This paper summarized the key sites at which α-glucosidase binds to some typical peptides, as shown in Figure 5.

## 5. Bioavailability of α-Glucosidase Inhibitory Peptides

Systemic delivery of functional peptides via the oral route has been a daunting task. Low bioavailability of peptide was due to denaturation in the digestive tract, susceptibility in enzymatic reaction, and poor permeability in the cell membrane [111]. In the current study, the bioavailability of peptide components was usually assessed via simulating gastrointestinal digestion. The stability of peptide bioactivity depends not only on structural maintenance but also on other factors [112]. Because the behavior of peptides that mimic the digestive species in the gastrointestinal tract can provide important information about their possible behavior in the digestive system. α-Glucosidase inhibiting peptides could exert hypoglycemic effects in vivo after entering the bloodstream in their active form. Therefore, peptides must withstand hydrolysis by gastrointestinal enzymes and continue to exert biological activity through the intestinal wall after oral administration [57]. Whether the peptide is consumed as a functional food ingredient or as a nutritional product, the ability to resist gastrointestinal digestion or interpretation is very important. In vitro simulated gastrointestinal digestion is a simple primary screening experiment that can be used to study the gastrointestinal degradation of bioactive peptides and then explore their bioavailability and bioactivity in vivo [113]. Two peptides WH and WS with strong α-glucosidase inhibitory activity were isolated from almond oil residue [74]. The two peptides were relatively stable under simulated digestion conditions. The inhibitory activity and structure of WH had no significant changes before and after digestion. The IC50 of α-glucosidase was 17.03 ± 0.05 μmol/Lol/L. The structure of WS did not change before and after digestion. The inhibitory activity of α-glucosidase was 44.63 ± 0.03 μmol/Lol/L and 24.71 μmol/Lol/L, respectively. Wei et al. [28] found the inhibitory capacity of YYFM and FFFM on α-glucosidase after digestion (65.76% and 52.90%, respectively) were lower than of undigested peptide (73.42% and 63.34%), but the inhibitory capacity of YFFM on α-glucosidase (37.2%) was higher than that of the undigested peptide (31.45%). These suggested that the inhibitory ability of the three peptides on α-glucosidase were relatively stable in the gastrointestinal digestive environment. Understanding the active efficacy of these peptides after gastrointestinal digestion is essential to assess their bioactivity and bioavailability. The resistances of active peptides in the gastrointestinal environment are mainly manifested in two aspects: first, these peptides themselves lack sites for cleavage by digestive enzymes; second, the combination and sequence arrangement of specific amino acid residues can interfere with the spatial structure or charged properties of the active sites, thus hindering the digestion. Some studies have suggested that peptides with lower molecular weight may have fewer protease recognition and cleavage sites; peptides resistant to gastrointestinal digestion have lower hydrophobicity and have more positive charge at pH 7.0 [114,115]. In addition, the effects of gastrointestinal digestion on the potency, stability, and bioavailability of α-glucosidase inhibitory peptides need to be further determined in vivo.

## 6. Conclusions

Foods not only provide nutrition, but also provide many functional ingredients that are beneficial to health. Food-derived α-glucosidase inhibitory peptides are non-toxic and can inhibit the increase in blood glucose concentration, which shows great potential in the management and treatment of diabetes. By systematically reviewing the research progress of α-glucosidase inhibitory peptides and analyzing the structural characteristics of α-glucosidase inhibitory peptides from different sources, it will be helpful to screen α-glucosidase inhibitory peptides more efficiently. Meanwhile, the discoveries of α-glucosidase inhibitory peptides can be accelerated by bioinformatics analysis. As dietary active peptides, it is also necessary to consider whether they will be degraded by digestive enzymes; thus, increasing the stability and bioavailability of α-glucosidase inhibitory peptides are important issues. Especially, will these bioactive peptides affect the gut microbiota and provide additional mechanisms for lowering blood glucose? These issues require further study. At present, there is still a long way to go for α-glucosidase inhibitory peptides use in the management and treatment of diabetes. Based on the existing research progresses, how can we develop α-glucosidase inhibitory peptides with therapeutic value? How can we cut the cost of preparing α-glucosidase inhibitory peptides? Can mixture peptides inhibiting α-glucosidase activity be used to lower blood glucose? These are the key issues to be solved in the future. In particular, the research on clinical trials needs to be strengthened and only obvious effect in population intervention experiments can be applied to food, health products, and drug development in the future.

## Figures and Tables

**Table 1:** Alpha-glucosidase inhibition of peptides and protein hydrolysates identified from different sources.

Source	Amino Acid Sequence	Interaction Mechanism		Inhibition (IC_50_)	Bound Residues of α-Glucosidase	References
		Model	Interaction			
Camel and bovine casein hydrolysates	LPTGWLM, MFE, GPAHCLL		Hydrogen bond		Trp376, Asp404, Ile441, Trp516, Asp518, Met519, Arg600, Trp613, Asp616, Phe649, His674;	[37]
Camel whey proteins	CCGM, MFE					[38]
Egg white protein	RVPSLM			23.07 μmol/L		[39]
	TPSPR			40.02 μmol/L		
Albumin	KLPGF			59.5 ± 5.7 μmol/L		[40]
	NVLQPS			100.0 ± 5.7 μmol/L		
Egg yolk protein hydrolysate	VTGRFAGHPAAQ			365.4 μg/mL		[27]
	LAPSLPGKPKPD			1065.6 mM		[41]
Parmigiano-Reggiano cheese	PFP			8.6 mmol/L		[42]
Fermented rubing cheese	QPHQPLPP		Hydrogen bonds	889 μmol/L	Arg428, Trp710, Asp568, Glu771, Asn448;	[43]
	TPVVVPPF			4330 μmol/L	Glu361, Glu443, Arg428;	
Silk cocoon hydrolysate	GEY			2.7 mg/mL		[44]
	GYG			1.5 mg/mL		
Silkworm pupae	QPGR		Hydrogen bonds	65.8 μmol/L		[45]
	SQSPA			20 μmol/L		
	QPPT			560 μmol/L		
	NSPR			205 μmol/L		
Sericin peptides	SEDSSEVDIDLGNLG	Noncompetitive	Hydrogen bonds; Van der Waals; Hydrophobic interaction	2.9 ± 0.1 μmol/L		[46]
Chinese giant salamander (*Andrias davidianus*) protein hydrolysate	CSSV			206 μg/mL		[47]
	YSFR			162 μg/mL		
	SAAP			66.9 μg/mL		
	PGGP			63.5 μg/mL		
	LGGGN			42.93 μg/mL		
Chinese giant salamander skin	GPPGPA	Competitive	Hydrogen bonds; Hydrophobic interaction		Hydrogen bonds: Asn53, Gln59, Trp80, Ala36, Ser59, Asp103, Gln102, Arg110, Arg188; Hydrophobic interaction: Ser56, Ala58, Asn199, Ser216, Leu78, Gln79, Phe225, Gln218, Ser18, Gln62, His66, Gln37, Tyr109, Leu393, Glu384, Lys106, Trp390, Ala211, Asp189, Tyr240, Ile185;	[48]
Edible insects	FDPFPK			5.95 μg/mL		[49]
Sardine muscle hydrolyzate	VW			22.6 mM		[50]
	WYPL			3.7 mM		
*Corbicula fluminea* and *Chlorella sorokiniana*	VKP, VKK, VW, WV, IW, LW					[51]
Soy protein	LLPLPVLK			237.43 ± 0.52 μmol/L		[52]
	SWLRL			182.05 ± 0.74 μmol/L		
	WLRL			165.29 ± 0.74 μmol/L		
*Ginkgo biloba* seed protein	LSMSFPPF		Hydrogen bonds; Van der Waals; Hydrophobic interaction	454.33 ± 32.45 μmol/L	Hydrogen bonds: Ala518, Pro512, His515, Phe516, Phe534, Ser92, Gly94; Hydrophobic interaction: Ala518, Ala533, Met535, Phe516, Phe534, Ala532, Val513, Ala451, Pro442; Van der Waals: Ala454, Phe455, Ala514, Asp440, Thr517, Thr519, Leu525, Asn447, Arg520, Leu93, His91, Lys89;	[53]
	VPKIPPP			943.82 ± 73.1 μmol/L	Hydrogen bonds: Pro230, Asn301, Asp333; Hydrophobic interaction: Ala343, Val335, Arg340, Met302, Ala232, Pro230, Phe397, Val334, Phe297; Van der Waals: Glu377, Thr339, Leu227, Leu300, Pro602, Gly228, Arg400, Tyr389, Glu396, Asn233, Ala229, Glu231;	
	MPGPPSD			1446.81 ± 66.98 μmol/L	Hydrogen bonds: Gly94, Asn4, Asp48, Arg457, Pro442, Asp440; Hydrophobic interaction: Phe463, Arg457, Lys96, Trp7; Van der Waals: Asp441, Asn447, Met6, Asp464, Lys483, Leu95, Val47, Asn46, Ala43, Arg456, His459, Pro460, Gly11;	
Soybean protein hydrolysate	GSR	Noncompetitive	Hydrogen bonds; Van der Waals; Anion-π interactions	20.4 μmol/L	Hydrogen bonds: Asp518, Asp616, Asp282; Van der Waals: Tyr292, Trp481, Asp518, Met519, Arg600, Trp613, Asp616;	[54]
	EAK			520.2 μmol/L	Hydrogen bonds: His674, Asp518, Arg600, Asp616, Asp282; Van der Waals: Trp376, Asp404, Ile441, Trp481, Trp516, Asp518, Phe649, His674; Anion-π interactions: Trp376, Trp481, Trp516, Phe649;	
Soft and hard wheat glutens	WGLYH		Hydrogen bonds; Electrostatic interaction			[55]
*Paeonia ostia* ‘Feng Dan’ seed protein	YFFM	Mixed-type	Hydrogen bonds; π-π stacking interactions		Hydrogen bonds: Tyr158, Asp352, Glu411, Arg315, Pro312; π-π stacking interactions: His280, Phe303;	[28]
	FFFM		Hydrogen bonds	245.46 ± 44.01 μmol/L	Hydrogen bonds: Asp307, Gly309, Thr310, Pro312, Leu313;	
	YYFM		Hydrogen bonds	306.71 ± 48.17 μmol/L	Hydrogen bonds: Tyr158, Ser240, Asp242, Asp352;	
Dark tea protein	TAELLPR			0.43 ± 0.03 mg/mL		[56]
	CGKKFVR			0.52 ± 0.09 mg/mL		
	AVPANLVDLNVPALLK			1.03 ± 0.13 mg/mL		
	VVDLVFFAAAK			0.04 ± 0.04 mg/mL	Pro395, Leu393, Val269, Glu271, Arg257, Phe297, Met281, Leu278, Asp322;	
Quinoa (*Chenopodium quinoa* Willd.)	IQEGGLT		Hydrophobic interaction			[57]
Sprouted quinoa yoghurt beverages	LAHMIVAGA	Noncompetitive	Hydrogen bonds	10.9 mg/mL		[58]
	VAHPVF			9.0 mg/mL		
Raw and cooked foxtail millet	AMFLPGA, TMMMLLP, FFLPQ, FMLPQ		Hydrogen bonds; Hydrophobic interaction		Hydrogen bonds: Arg600, Arg281, Ala284, Asp518, Ser676, Gly651, Trp481, His674; Hydrophobic interaction: Trp376, Trp481, Trp681;	[59]
Fermented rice bran	GLLGY	Noncompetitive	Hydrogen bonds		Asp282, Ser523, Asp616, His674;	[25]
Hemp (*Cannabis sativa* L.) seed protein	LR, PLMLP		Hydrophobic interaction			[60]
The roots of *Gypsophila oldhamiana*	Cyclo-(GFDFILP)			305 μmol/L		[26]
Wheat germ peptides	LDLQR		Hydrogen bonds	8.59 mM	Asp443, Arg334, Gln603, Asp203, Asp542;	[61]
	AGGFR			8.66 mM	Asp542, Gln603	
	LDNFR			9.21 mM		
*Spirulina platensis*	GVPMPNK			151.5 μg/mL		[62]
	RNPFVFAPTLLTVAAR			164.5 μg/mL		
	LRSELAAWSR			134.2 μg/mL	Thr168, Leu144, Ile146, Gly149, Arg10, Trp8;	
Walnut (*Juglans mandshurica* maxim.)	LPLLR					[63]
Hot-pressed peanut meal protein	FYNPAAGR, PGVLPVAS, FFVPPSQQ, FSYNPQAG		Hydrogen bonds; Hydrophobic interaction; Salt bridges; π-stacking		Hydrogen bonds: Arg202, Asp203, Thr205, Asn209, Asp327, Asp443, Asp474, Arg526, Asp542; Hydrophobic interaction: Thr204, Tyr299, Trp406, Phe450, Leu473, Lys480, Phe575; Salt bridges: Asp327, Asp443, Asp542, Arg202;	[64]
Black bean (*Phaseolus vulgaris* L.)	TTGGKGGK		Hydrogen bonds; Hydrophobic interaction; Polar interactions			[65]
	AKSPLF				Hydrogen bonds: Asp34, Thr83, Asp89; Polar interactions: Asp34, Thr83, Asp89, Asn32;	
	QTPF				Hydrogen bonds: Asn32, Asp34, Thr83; Polar interactions: Asn32, Asp34, Trp36, Thr83;Hydrophobic interaction: Pro82;	
	FEELN				Hydrogen bonds: Asp34, Thr83; Polar interactions: Asn32, Asp34, Thr83;	
	LSKSVL				Hydrogen bonds: Asp34, Thr83, Asp89, Asn32; Polar interactions: Asp34, Thr83, Asp89, Asn32;	
Chickpea (*Cicerarietinum* L.) protein hydrolyzates	FGKG	Competitive	Hydrophobic interaction		Trp59, Leu162, Ala198, His305;	[66]
Camellia seed cake	LLVLYYEY	Noncompetitive	Hydrogen bonds	0.33 mM	Arg730, Gly732, Arg653, Glu661;	[67]
	LLLLPSYSEF	Mixed-type		1.11 mM	Asp203, Thr205, Tyr605, Gln603;	
*Aspergillus oryzae*	PFP	Mixed-type		3.1 mg/mL		[68]
*Pseudomonas fluorescens* IB-MR-66e	Cyclo(_L_-Pro-_L_-Leu)		Hydrophobic interaction; Hydrophilic interaction; Hydrogen bonds		Hydrogen bonds: Ser157; Hydrophobic interaction: Lys156, Tyr158, Asp242, Val232, Asp233, Phe314, Arg315, Tyr316, Asn415; Hydrophilic interaction: Ser157, Ser240, Ser241;	[69]
Soft-shelled turtle egg	ARDASVLK		Hydrogen bonds; Hydrophobic interaction	195 μmol/L	Tyr158, Ser240, Asp242, Glu277, Thr310, Pro312, Arg315, Asp352, Glu411;	[70,71]
	HNKPEVEVR			56 μmol/L	Lys156, Ser157, Asp242, Asp307, Pro312;	
	SGTLLHK			289 μmol/L	Asp215, Glu277, Asp307, Asp352;	
*Moringa oleifera* seed protein	KETTTIVR		Hydrogen bonds; Hydrophobic interaction	109.65 μmol/L	Hydrogen bonds: Arg429, Asp379, Val380, Asp333, Leu300, Leu227, Lys398; Hydrophobic interaction: Ala378, Gly399, Arg400, Phe397, Val334, Tyr389, Asn301, Ala229, Met302, Val335, Glu231, Pro230, Pro395, Glu377;	[72]
Lupin protein hydrolysate	SPRRF, FE, RR, RPR, LRP, PPGIP		Hydrogen bonds; Hydrophobic interaction		Hydrogen bonds: Asp616, Asp518, Arg411; Hydrophobic interaction: Trp376, Phe649, Asp404, Asp616, Ser676, Leu650, Leu678, Trp481, Asp282, Phe525, Phe649, Trp613, Leu677, Ser379, Asn417, Leu405, Asp419, Met408, Ser410, Arg600, Met519;	[73]
Almond (*Armeniaca sibirica*) oil manufacture residue	WH			16.99 ± 0.05 μmol/Lol/L		[74]
	WS			44.63 ± 0.03 μmol/Lol/L		

Amino acid nomenclature: C, cysteine; H, histidine; I, isoleucine; M, methionine; S, serine; V, valine; A, alanine; G, glycine; L, leucine; P, proline; T, threonine; F, phenylalanine; R, arginine; Y, tyrosine; W, tryptophan; D, aspartic acid; N, asparagine; E, glutamic acid; Q, glutamine; K, lysine.

## Abbreviations

ACE	Angiotension Converting Enzyme
DPP-IV	Dipeptidyl Peptidase IV
ESI-MA	Electrospray Ionization Mass Spectrometry
GDM	Gestational Diabetes Mellitus
IC_50_	Half Maximal Inhibitory Concentration
LC-MS	Liquid Chromatography–Mass Spectrometry
MW	Molecular Weight
MS	Mass Spectrum
MALDI-TOF	Matrix-Assisted Laser Desorption Time-of-Flight Mass Spectrometry
Q-TOFMS	Time-of-Flight Tandem Mass Spectrometer
RP-HPLC	Reverse Phase–High Performance Liquid Chromatography
SEC	Size Exclusion Chromatography
SDS-PAGE	Sodium Dodecyl Sulfate-Polyacrylamide Gel Electrophoresis
T1DM	Type 1 Diabetes Mellitus
T2DM	Type 2 Diabetes Mellitus
